# Significance of Spliceosome-Related Genes in the Prediction of Prognosis and Treatment Strategies for Lung Adenocarcinoma

**DOI:** 10.1155/2022/1753563

**Published:** 2022-11-04

**Authors:** Ying Yang, Tianyi Huang, Yihui Fan, Haimin Lu, Jingjing Shao, Yilang Wang, Aiguo Shen

**Affiliations:** ^1^Cancer Research Center Nantong, Nantong Tumor Hospital, The Affiliated Tumor Hospital of Nantong University, Nantong University, Nantong, China; ^2^Department of Pharmacy, Nantong University Xinling College, Nantong, China; ^3^Department of Thoracic Surgery, Nantong Tumor Hospital, The Affiliated Tumor Hospital of Nantong University, Nantong University, Nantong, China; ^4^Department of Oncology, Nantong Tumor Hospital, The Affiliated Tumor Hospital of Nantong University, Nantong University, Nantong, China

## Abstract

**Background:**

The leading cause of cancer-related fatalities globally is lung cancer; lung adenocarcinoma (LUAD) is the most common histological type in it. The spliceosome plays an important role in a majority of malignancies. However, it is yet unclear how spliceosome-related genes affect patients with LUAD in terms of treatment course and prognosis.

**Methods:**

Spliceosome-related genes were assessed from The Cancer Genome Atlas (TCGA) and Gene Expression Omnibus (GEO) database to obtain clinical information and gene expression in patients with LUAD. A spliceosome-related gene signature and prognostic model were constructed by using the least absolute shrinkage and selection operator (LASSO), time-dependent receiver operating characteristic (ROC), and nomogram. Immune infiltrate levels, mutation analysis, and pathway enrichment were predicted potential mechanisms of the signature by using single-sample gene set enrichment analysis (ssGSEA), Gene Set Cancer Analysis (GSCA) database, Kyoto Encyclopedia of Genes and Genomes (KEGG), and Gene Ontology (GO) database. Then, a protein–protein interaction (PPI) network and transcription factor- (TF-) hub gene and drug mining network were also established by Cytoscape software.

**Results:**

Firstly, we constructed a prognostic model for 11 spliceosome signature genes. Based on the prognostic risk score, we stratified patients with LUAD into high- and low-risk groups. The high- and low-risk groups were closely related to the OS, tumor immune infiltration level, immune checkpoint molecules, and tumor mutation burden (TMB) of LUAD patients. Based on PPI networks, we also predict relevant TF genes that may regulate signature prognostic genes. Finally, drugs including oxaliplatin, arsenic trioxide, cisplatin, and sunitinib were excavated for the treatment of the 11 spliceosome signature genes in LUAD patients.

**Conclusion:**

In conclusion, this study is the first to explore the importance of spliceosome-related genes in the prognosis and treatment of LUAD. Through our study, we have innovatively provided potential prognosis genes and new therapeutic drug targets for the treatment of LUAD patients.

## 1. Introduction

The majority of cancer-related deaths globally are caused by lung cancer. According to estimates, there will be 2206771 new cases of lung cancer (11.4% of all sites) and 1796144 deaths (18.0% of all sites) in 2020 [1]. Based on its histology, non-small-cell lung cancer (NSCLC) is the most significant type of lung cancer, accounting for about 85% of total cases. Among them, NSCLC is mainly divided into lung adenocarcinoma (LUAD) and lung squamous cell carcinoma (LUSC) [2]. LUAD is the most common subtype of NSCLC, and most patients with LUAD were diagnosed at an advanced stage [3]. LUAD patients increase survival through surgery, chemotherapy, radiotherapy, targeted treatment, and immunotherapy. However, due to being prone to recurrence, metastasis, and drug resistance after treatment, the five-year mortality rate for lung cancer patients is greater than 80% [4]. Therefore, it is crucial to look for novel, essential target genes in order to improve treatment options and patient outcomes for LUAD patients.

The spliceosome is a multi-subunit RNA–protein complex that catalyzes the splicing of nuclear precursor mRNA (pre-mRNA) into mRNAs [5]. The spliceosome mainly contains five small nuclear ribonucleoproteins (snRNPs), and each of the five snRNPs (U1, U2, U4, U5, and U6) consists of an snRNA molecule, seven Sm proteins, and other snRNP-specific factors [6, 7]. Previous studies have found that U1, U2, U4, and U5 snRNP share the Sm heptamer ring, named the “fixed” Sm ring. In contrast, the heptamer ring in U6 snRNP contains seven Sm (Lsm)-like proteins known as “flexible” Sm rings [8, 9]. These molecules are involved in the splicing of pre-mRNA, including snRNPs and Lsm proteins. Previous studies have found that snRNPs, as spliceosome-related proteins, are involved in abnormal cell biological activities, including melanocytoma, triple-negative breast cancer, and hepatocellular carcinoma [10, 11]. To date, there have been fewer studies that have found that the Sm protein, which forms a ring structure in the core of the spliceosome, is a selective lethal target for cancer. Interference with Sm protein expression can induce apoptosis of NSCLC cells, but not nonmalignant cells [12, 13]. Additionally, it is unclear exactly how the LSM protein affects NSCLC. The prognosis, course of treatment, and role of spliceosome-related genes in LUAD patients are not yet fully understood.

In this study, we thoroughly examined the function of snRNPs and LSMs in LUAD from a bioinformatics approach. To predict the prognosis and available treatments for LUAD, we set out to construct a gene-based signature from spliceosome-related genes. We used multiple datasets to mainly analyze the relationship between spliceosome-related genes and prognostic risk models, immunotherapy, and drug mining. In conclusion, it is the first to explore the importance of spliceosome-related genes in the prognosis and treatment of LUAD. Through our study, we have innovatively provided potential prognosis genes and new therapeutic drug targets for the treatment of LUAD patients.

## 2. Materials and Methods

### 2.1. Data Acquisition and Preprocessing

**Figure 1:**
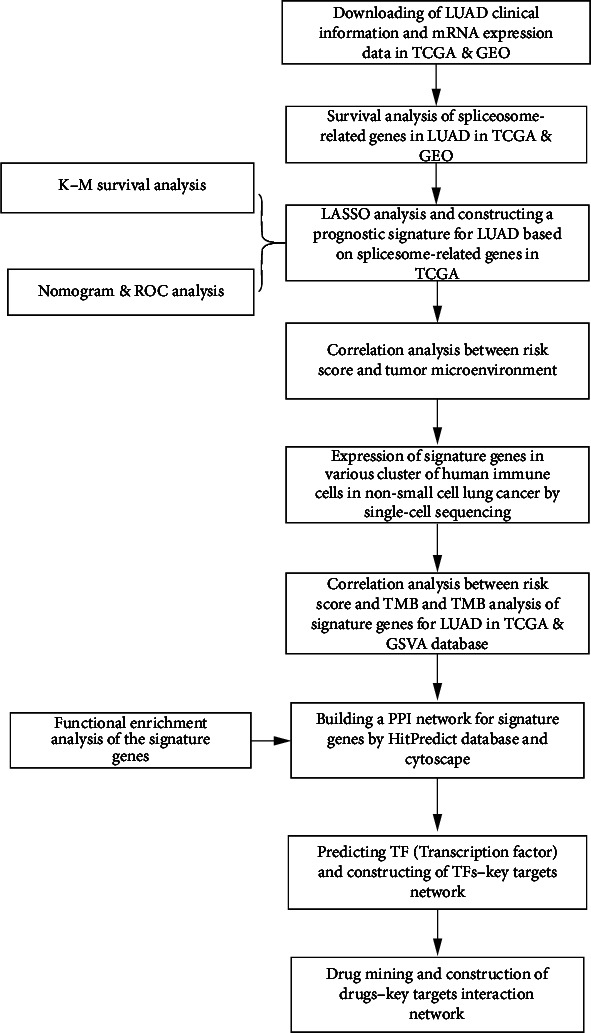
The flowchart for research design.

The research design and procedures are overviewed in Figure 1. We download standardized FPKM gene expression profiling data and clinical samples for LUAD from TCGA database (https://portal.gdc.cancer.gov). Among the tumor samples, 500 samples with clinical survival and prognostic information were retained as the training set. And tumor samples with no clinical survival information and incomplete or zero prognosis time were excluded. Furthermore, the GSE10072 and GSE42127 gene expression profiling datasets were obtained from the Gene Expression Omnibus (GEO) database (http://www.ncbi.nlh.gov/gds), which was used to verify the expression levels and survival analyses of differential genes.

### 2.2. Gene Expression Analysis

500 tumor samples and 59 normal samples that complied with the criteria were included in the TCGA-LUAD database to analyze the expression variations in spliceosome-related genes (SNRPB/D1/D2/D3/E/F/G/N, LSM1-8) between tumors and adjacent normal tissues. In addition, we verified the outcomes of these genes' differential expression using the GSE10072 dataset.

### 2.3. Survival Prognosis Analysis

We investigated the overall survival (OS) statistics for the 16 genes associated with spliceosome in LUAD by TCGA database and validated them in the GSE42127 dataset. The data was graphically shown with the Survival R tool. All of the aforementioned diagrams were constructed using the Sangerbox database (http://www.sangerbox.com/).

### 2.4. Prognostic Signature Construction

Based on the differential expression and survival prognosis value of 16 spliceosome-related genes, the LASSO regression model was constructed by using the R software package “glmnet.” In addition, the 10-fold cross-validations were set up to get the optimal model. Finally, the spliceosome-related gene signature was established by using multivariate Cox regression analysis and a risk score for each LUAD patient was calculated by the following formula:

(1) $$\begin{array}{c} \text{risk score}=\overset{n}{\underset{i=1}{\sum }}\left(\beta_{i}*\text{geneExp}i\right). \end{array}$$

A prognostic heat map analysis was performed to assess the relationship between high- and low-risk score and survival prognosis of these 11 signature genes. The nomogram and ROC curve were established to evaluate the survival prediction accuracy of risk score model by using the R package.

### 2.5. Functional Enrichment Analysis

Kyoto Encyclopedia of Genes and Genomes (KEGG) and Gene Ontology (GO) enrichment analyses were performed using the R package “clusterProfiler” (version 3.14.3) to obtain the enrichment results for the snRNPs and LSM genes. *P* < 0.05 was considered statistically significant in the enrichment results.

### 2.6. Immune Infiltration and Tumor Mutation Burden Analysis

The risk score is divided into high-risk groups and low-risk groups according to the median. Estimation of STromal and Immune cells in MAlignant Tumors using Expression data (ESTIMATE) is a computational tool that predicts tumor purity and consists of three main scores: stromal score, immune score, and estimate score. Visualizations of violins were used to compare the correlation between immune cell infiltration levels and risk scores. We performed a single-sample gene collection enrichment analysis (ssGSEA) using the “gene set variation analysis (GSVA)” R package and quantified infiltration of 28 immune cell types based on the risk score.

We explored the correlation between gene expression and single-cell global through a single-cell sequencing database by using Single Cell Portal (https://singlecell.broadinstitute.org/single_cell). Using a public database of single-cell sequencing of NSCLC (http://lung.cancer-pku.cn/index.php), tSNE was used to analyze the global characterization of 11 key genes in NSCLC single-cell sequencing T cells.

We download the mutation information of LUAD samples through TCGA database and use the “maftool” R package to calculate the tumor mutation burden (TMB) of tumor samples. The risk score was divided into high and low groups, and the correlation between TMB score and risk score was compared. Through the Gene Set Cancer Analysis (GSCA) database (http://bioinfo.life.hust.edu.cn/GSCA/#/), we searched the mutation status of 11 signature genes in LUAD.

### 2.7. Protein–Protein Interaction of the Signature Genes

We use the HitPredict database (http://www.hitpredict.org/) to search the protein interactions of 11 signature genes. The protein interaction network of the snRNP genes and the LSM genes is plotted separately using Cytoscape software, and the hub genes were presented according to the “degree” value.

### 2.8. Construction of Transcription Factor-Signature Gene Network and Mining of Chemical Drug-Signature Gene Interaction Network

The regulatory network of hub genes and LUAD was built using Cytoscape software. Then, the plugin iReculon is used to predict transcription factors (TF) to regulate the network. The potential chemical drugs that interacted with 11 signature genes were predicted using the Comparative Toxicogenomics Database (CTD) (http://ctdbase.org/). Then, the potential drug-hub gene interaction network was constructed through Cytoscape software. In particular, chemotherapy and targeted drugs that have been used extensively in clinical settings may interact with at least two target genes.

## 3. Results

### 3.1. Expression Analysis of Spliceosome-Related Genes in LUAD

**Figure 2:**
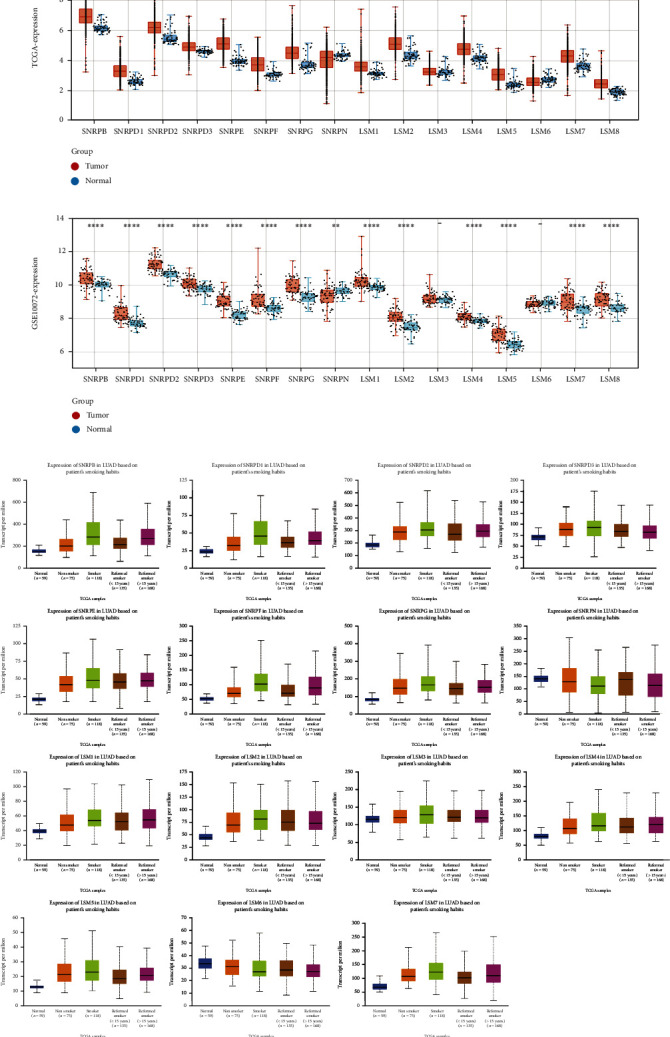
Expression analysis of spliceosome-related genes in LUAD. (a) The differential expression of spliceosome-related genes in LUAD and normal samples from TCGA database. (b) The differential expression of spliceosome-related genes in LUAD and normal samples from the GEO database (GSE10072). (c) The relationship between spliceosome-related gene expression and smoking habits in LUAD and normal patients from the UALCAN database (^∗^, ^∗∗^, and ^∗∗∗^ represent P < 0.05, P < 0.01, and P < 0.001, respectively).

To explore the differential spliceosome gene expression in LUAD and adjacent normal samples, we obtained normalized gene expression data from TCGA and GEO databases. In TCGA-LUAD database, all spliceosome-related genes showed significant differential expression (except LSM3) (Figure 2(a)). In the validation cohort, we found significant differences in the expression of spliceosome genes except for LSM3 and LSM6 (Figure 2(b)). In addition, using the UALCAN database, we analyzed the relationship between spliceosome-related gene expression and smoking habit in LUAD and normal samples and found that 16 genes showed significant differences in it (*P* < 0.05) (Figure 2(c)).

### 3.2. The Prognostic Value of Spliceosome-Related Genes in LUAD

**Figure 3:**
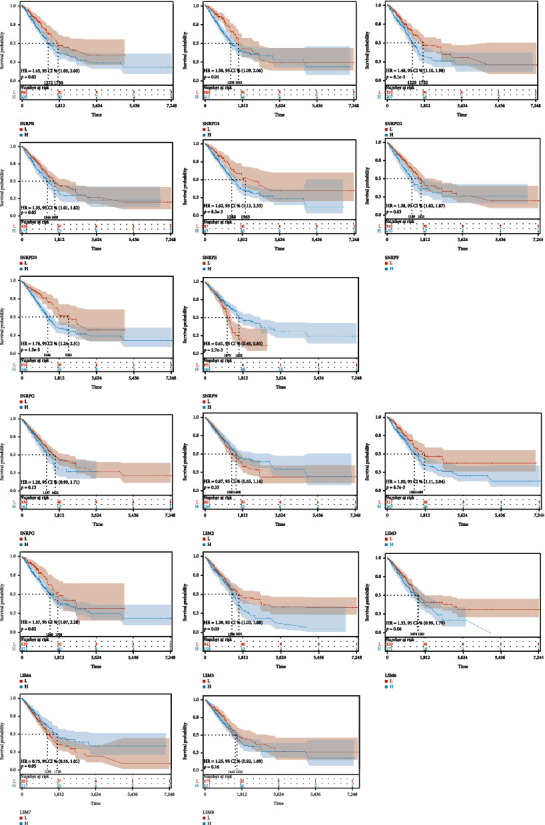
The overall survival of spliceosome-related genes of LUAD in TCGA. The Kaplan–Meier (KM) survival analysis of the spliceosome-related genes in the TCGA-LUAD database as training cohort (P < 0.05 represents significance).

**Figure 4:**
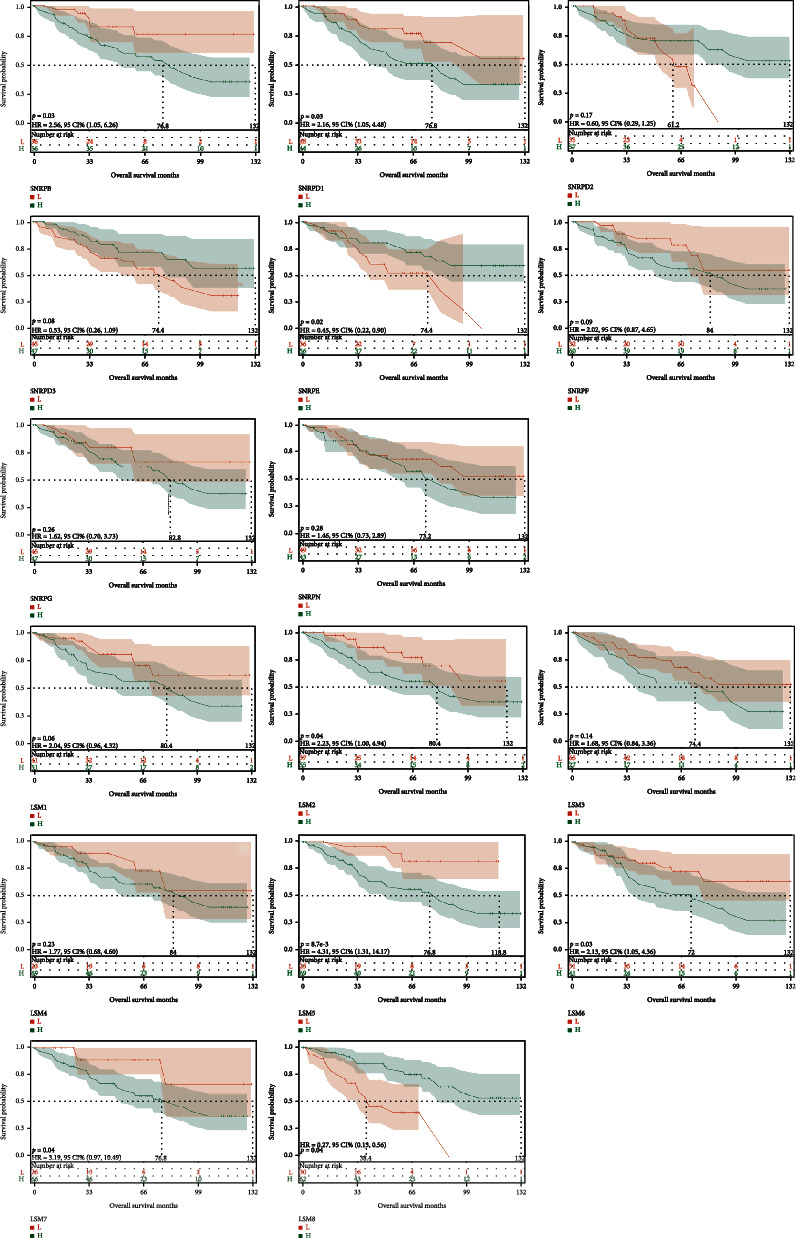
The overall survival of spliceosome-related genes of LUAD in the GEO database. The KM survival analysis of the spliceosome-related genes in GSE42127 as validation cohort (*P* < 0.05 represents significance).

In the training cohort of TCGA-LUAD, we found that high expressions of LSM3, LSM4, LSM5, SNRPB, SNRPD1, SNRPD2, SNRPE, SNRPF, and SNRPG were all associated with poor prognosis, while the prognostic trend of SNRPN was reversed (Figure 3). In the validation cohort of GSE42127, we found that the high expressions of LSM2, LSM5, LSM6, LSM7, SNRPB, and SNRPD1 were all associated with poor prognosis, while the prognostic trend of LSM8 and SNRPE was reversed (Figure 4).

### 3.3. Construction and Validation of a Prognostic Signature for LUAD

**Figure 5:**
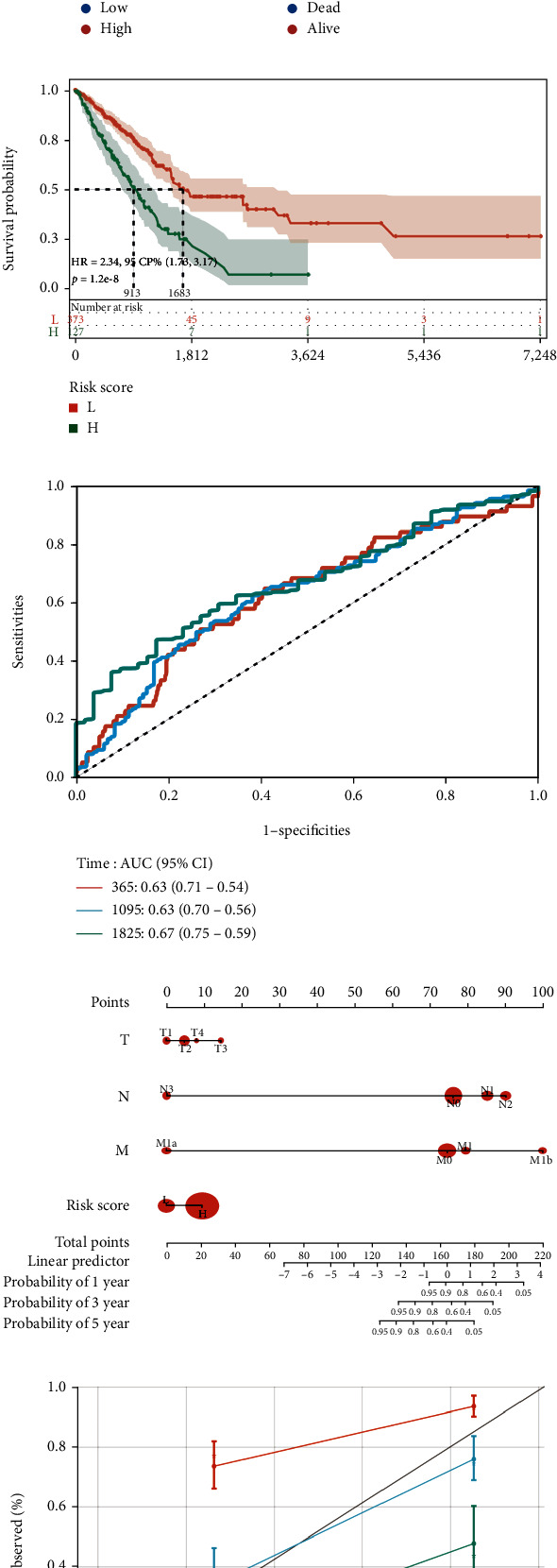
Construction and validation of a prognostic signature for LUAD. (a, b) The prognostic model of 11 spliceosome-related genes was constructed using LASSO regression analysis. (c) Based on the high- and low-risk groups, the prognostic risk heat map displayed the survival events in LUAD patients and changes in the signature gene expression. (d, e) The KM survival and ROC curve analysis based on the high- and low-risk groups. (f, g) The prognostic nomogram and validation curve in LUAD patients. (h, i) The PAC and tSNE analysis based on the high- and low-risk groups (*P* < 0.05 represents significance).

We discovered that there were no appreciable variations in the OS of several genes based on the aforementioned survival analyses. However, not all spliceosome-related genes expressed differently can precisely forecast the OS of individuals with LUAD. Therefore, we attempted to obtain the genetic signature from the 16 genes related to spliceosome. Finally, we constructed a LASSO regression model based on spliceosome-related gene expression and prognosis from 500 LUAD patients. Based on dimensionality reduction analysis of the LASSO regression model, we finally obtained 11 key signature genes (SNRPB, SNRPE, SNRPF, SNRPG, SNRPN, LSM2, LSM4, LSM5, LSM7, and LSM8) (Figures 5(a) and 5(b)).

The following formula is used to calculate the risk score for each patient in LUAD: RiskScore = 0.0387730558334106^∗^SNRPB + 0.222163252536089^∗^SNRPD2 + 0.316667345957381^∗^SNRPE + 0.00102350161032576^∗^SNRPF − 0.0273995193485904^∗^SNRPG − 0.104560226441857^∗^SNRPN − 0.332380917177904^∗^LSM2 + 0.0859569595128913^∗^LSM4 + 0.170065628325337^∗^LSM5 − 0.102801853840285^∗^LSM7 − 0.0896630619670466^∗^LSM8.

Calculating the risk score based on the prognostic risk model, we analyzed the prognostic heat map about survival events of patients and expression changes of signature genes. Then, we observed that the survival rate of patients decreased significantly as the risk score increased (Figure 5(c)). According to the optimal truncation value of the risk score, the LUAD samples were divided into high- and low-risk groups and KM analysis was carried out. The results revealed significant variations between these two groups, and the patient's prognosis was negatively correlated with risk score (Figure 5(d)). The ROC curve showed that the prognostic profile has satisfactory predictive accuracy for patients with LUAD, with an AUC of 0.63 at 1 year, 0.63 at 3 years, and 0.67 at 5 years (Figure 5(e)).

Following that, we used TCGA training cohort to create a nomogram based on risk score and TNM (Figure 5(f)). The *C* index of line chart is around 0.730, which showed that it performed well in terms of prediction. The dependability and correctness of the line chart were confirmed by calibration curve analysis, which showed that the predicted probabilities of the 1-year, 3-year, and 5-year OS were optimally comparable with the actual observations (Figure 5(g)). Finally, when the samples were categorized based on their risk score, PCA and t-SNE revealed that the high- and low-risk groups had various distribution trajectories (Figures 5(h) and 5(i)).

### 3.4. Functional Enrichment Analysis

**Figure 6:**
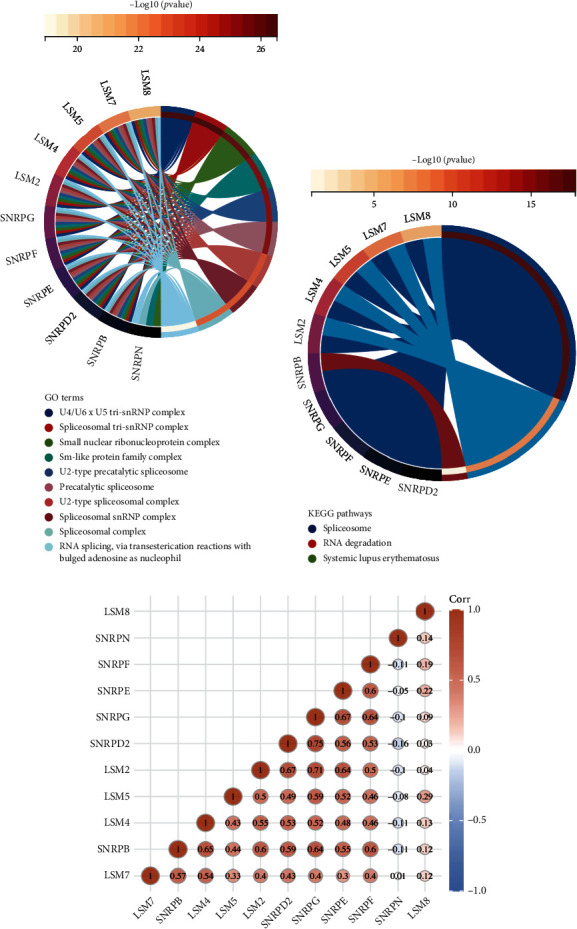
Functional enrichment analysis of spliceosome signature genes. (a, b) GO and KEGG analysis of spliceosome-related genes. (c) The correlation analysis of spliceosome signature genes.

To analyze the pathway associated with the signature genes, GO and KEGG enrichment analyses were performed. The results of GO analysis showed that both the snRNPs and LSM genes were mainly closely related to pathways such as small ribosome protein complexes, Sm-like protein family complexes, splicer complexes, RNA splices, and applicant snRNP assembly (Figure 6(a)). KEGG analysis showed that these genes were associated with applicants, RNA degradation, and systemic lupus erythematosus (Figure 6(b)).  Figure 6(c) shows the correlation analysis of 11 signature genes and found that SNRPD2 most strongly correlated with SNRPGs (correlation coefficient: 0.75).

### 3.5. Spliceosome Signature Genes Predict Immune Cell Infiltration

**Figure 7:**
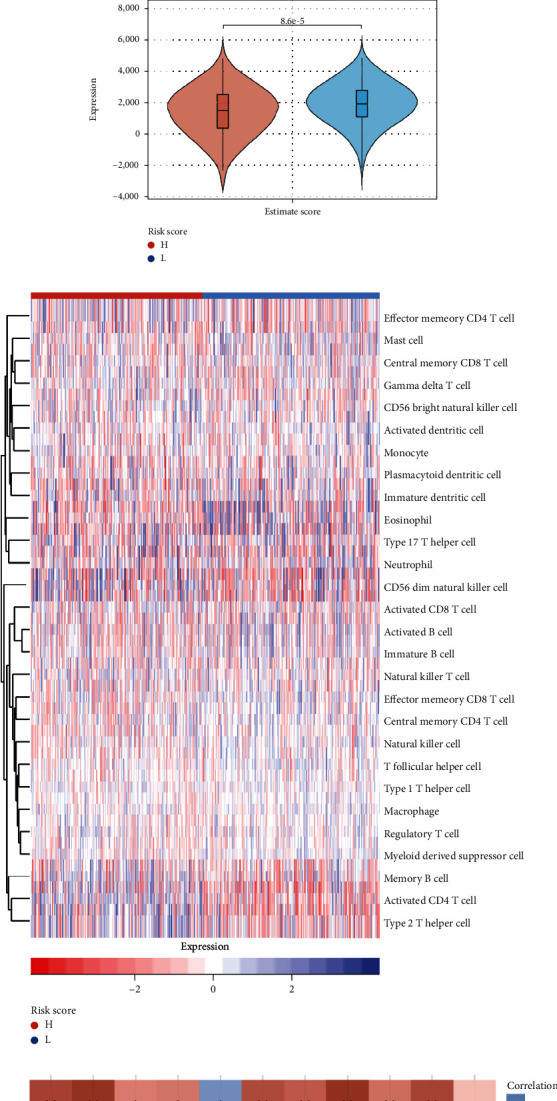
Spliceosome signature genes predict the level of immune cell infiltration. (a–c) The violin chart showed the comparison of stromal score, immune score, and ESTIMATE score. (d) The correlation of risk score with 28 immune infiltration levels. (e) The relationship between immune checkpoints and spliceosome signature genes (^∗^, ^∗∗^, and ^∗∗∗^ represent P < 0.05, P < 0.01, and P < 0.001, respectively).

We analyzed the variations in ESTIMATE immunological ratings across high-risk and low-risk groups in order to investigate the relationship between risk scores and tumor purity. The findings demonstrated that the high-risk groups consistently displayed low levels of immune infiltrates in the stromal score, immunological score, and estimation score value (Figures 7(a)–7(c)).

As shown in Figure 7(d), there were differences in immune infiltration levels between the high- and low-risk groups. Notably, cell subtypes such as memory B cells, activated CD4 T cells, and Th2-type helper T cells showed lower infiltration abundance in the high-risk group.  Figure 7(e) shows an analysis of the correlation between 11 signature genes and immune checkpoints. From the figure, it was not difficult to find that most of the spliceosome-related genes were significantly related to immune genes. In particular, SNPRE showed a significant negative correlation with TIGIT, SIGLED15, PDCD1LG2, PDCD1, LAG3, HAVCR2, CTLA4, and CD274.

**Figure 8:**
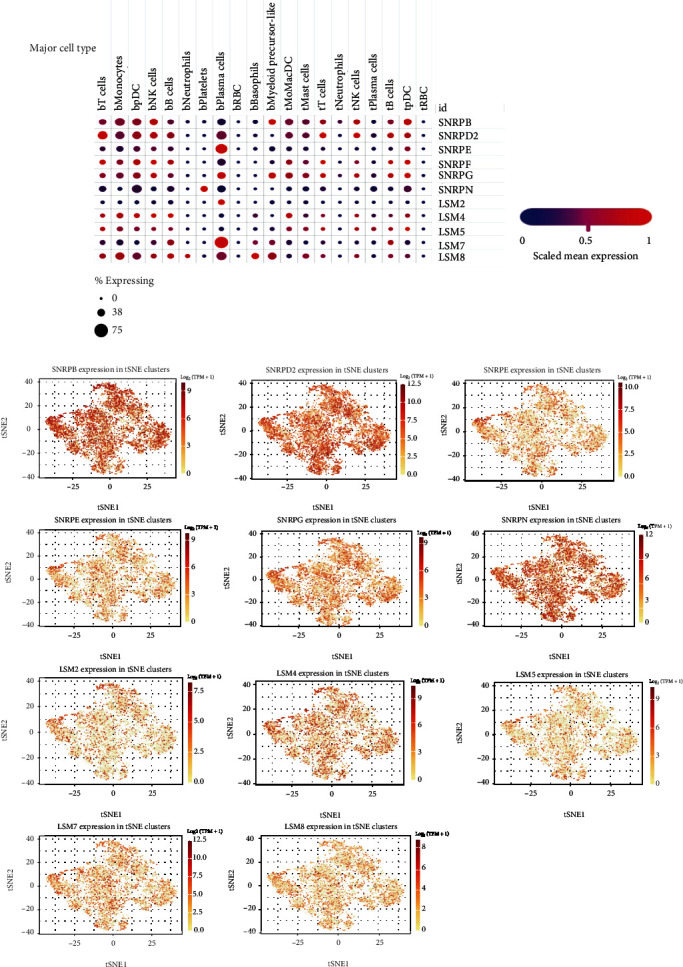
Expression of spliceosome signature genes in single-cell sequencing of NSCLC. (a) Expression of spliceosome signature genes in human immune cell of NSCLC (t: tumor; b: peripheral blood; DCs: dendritic cells; pDCs: plasmacytoid DCs; RBC: red blood cell). (b) The global characterization of 11 signature gene analysis in NSCLC single-cell sequencing T cells.

To further explore the correlation between these 11 signature genes and immune cells, we explored the correlation between these 11 signature gene expressions and single-cell global through a single-cell sequencing database. In order to identify the expression of these 11 signature genes in various immune cell types, we used Single Cell Portal to plot myeloid cells from NSCLC peripheral blood and tumor tissue. As shown in Figure 8(a), 11 genes were expressed in lung cancer cell subsets, of which SNRPD2, SNRPE, LSM7, and LSM8 have significantly higher expression in lung cancer peripheral plasma cells. By using a public database of single-cell sequencing of NSCLC, tSNE figures were used to analyze the global characterization of 11 signature genes in NSCLC single-cell sequencing T cells (Figure 8(b)).

### 3.6. Analysis of Mutation in Spliceosome Signature Genes

**Figure 9:**
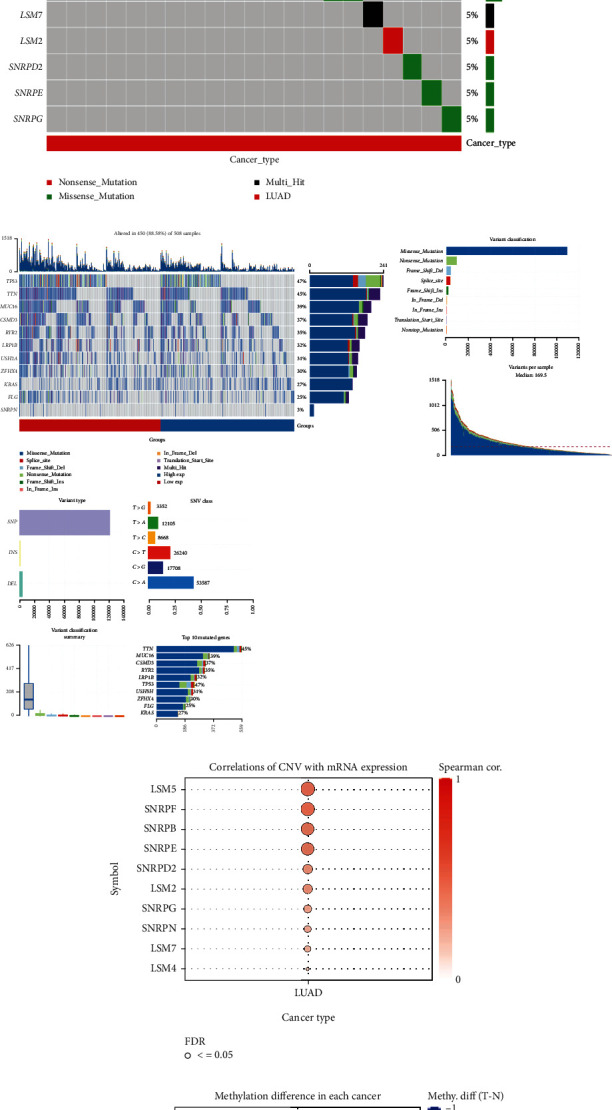
Analysis of mutation in spliceosome signature genes. (a) The correlation between TMB and risk score. (b) The mutation frequency of 11 spliceosome signature genes in the high- and low-risk groups. (c) A genetic mutation landscape analysis. (d) The correlation between CNV and spliceosome signature gene mRNA expression. (e) The methylation difference of spliceosome signature genes in LUAD (*P* < 0.05 represents significance).

As shown in Figure 9, we performed mutation and CNA analysis on 11 spliceosome-related genes. Firstly, we compared the correlation between TMB and risk score, and the results showed that the TMB values were lower in the high-risk group (Figure 9(a)). From Figure 9(b), it showed that SNRPN had the highest mutation rate among the 11 genes based on the high- and low-risk groups. In addition, a genetic mutation landscape analysis was performed on SNPRN, as shown in Figure 9(c). By conducting through the GSCA database, it showed the correlation between CNV and mRNA expression in 11 signature genes, with LSM5 being the most significantly correlated (Figure 9(d)).  Figure 9(e) shows the correlation between 11 genes and methylation, and SNRPB showed a significant correlation with methylation in LUAD.

### 3.7. Protein–Protein Interaction of the Signature Genes

**Figure 10:**
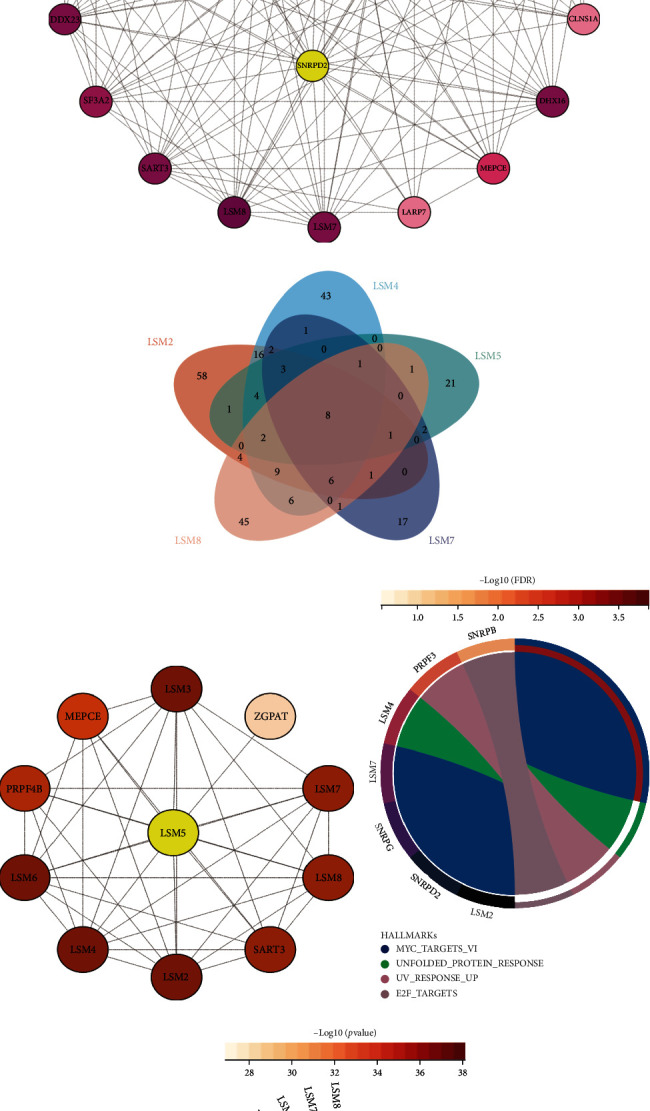
Protein–protein interaction of the spliceosome signature genes. (a) Venn diagrams showed proteins interacting with snRNP signature genes based on the HitPredict database. (b) The PPI network map for snRNP signature genes by Cytoscape software. (c) Venn diagrams showed proteins interacting with LSM signature genes based on the HitPredict database. (d) The PPI network map for LSM signature genes by Cytoscape software. (e) The hallmark analysis based on PPI network. (f) The GO pathway enrichment analysis.

Using the HitPredict database, we searched for interaction proteins about these 11 spliceosome signature genes. Figures 10(a) and 10(b), with 18 genes of SNRPB, SNRPE, SNRPPF, SNRPG, and SNRPN interacting together using the Venn diagram, demonstrate the protein interaction network of snRNPs by Cytoscape software. According to the degree value, SNPRB, SNPRD2, and SNRPN were at the center of the network diagram. From Figures 10(c) and 10(d), the Venn diagram was used the same to obtain LSM2, LSM4, LSM5, LSM7, and LSM8 interacting 8 genes, to plot the protein interaction network. According to the degree value, LSM5 is located at the center of the network. Functional enrichment analysis was performed by combining all proteins that interact with spliceosome-related genes (Figures 10(e) and 10(f)). The KEGG hallmark results showed that these molecules were mainly related to MYC, E2F, and other signaling pathways. GO analysis is almost identical to Figure 6(a).

### 3.8. Construction of Transcription Factor-Signature Gene Network and Mining of Chemical Drug-Signature Gene Interaction Network

**Figure 11:**
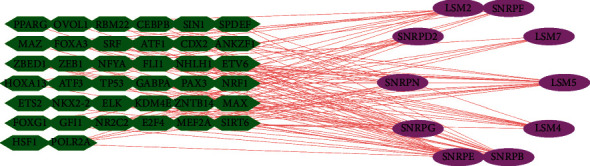
Construction of transcription factor-signature gene network. A Cytoscape software to establish a transcriptional regulatory network of hub genes and TFs (the green node represents TFs, and the purple node represents targets).

To enhance the clinical value and significance of our study, we utilized Cytoscape software to establish a transcriptional regulatory network of hub genes and TFs. As shown in Figure 11, a total of 38 TFs and 11 hub genes were involved in the network.

**Figure 12:**
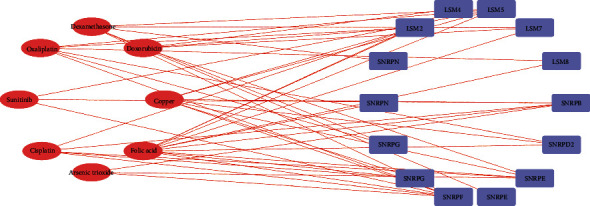
Mining of chemical drug-signature gene interaction network. Interaction between drugs and the spliceosome signature genes based on CTD database (the red node represents chemical drugs, and the purple node represents spliceosome signature gene).

We used CTD databases to identify pharmaceuticals that precisely target 11 pivot genes, allowing us to offer a more precise and individualized selection of tailored medications for LUAD clinical management. Potential drugs that interact with at least two pivot genes and have been shown to have anticancer pharmacological effect must meet the inclusion criteria for targeted drugs. Finally, eight drugs are included, including cisplatin, doxorubicin, dexamethasone, oxaliplatin, sunitinib, and copper (Figure 12). Therefore, there is an urgent need to identify new biomarkers and study molecular mechanisms.

## 4. Discussion

Lung cancer is the most common malignancy worldwide. According to the global cancer data released by the WHO in 2021, the incidence and mortality rate of lung cancer in China rank first in the world. Although lung cancer treatments have diversified in recent years, the 5-year survival rate for patients remains low [14]. As one of the most important subtypes of NSCLC, patients are in the middle and advanced stages of the disease when they are diagnosed [3, 15]. Posttreatment recurrence, metastasis, and drug resistance significantly reduce the quality of survival in patients with LUAD. Therefore, there is an urgent need to explore new biomarkers and target genes to provide new treatment strategies and improve prognostic value for LUAD patients.

In this study, we predicted the molecular mechanisms of LUAD in TCGA and GEO databases from a bioinformatics perspective. We used LASSO regression model to establish a novel prognostic signature based on spliceosome-related genes in LUAD. Additionally, the relationship between tumor microenvironment, tumor mutation burden, and 11 signature gene prognostic risk models were compared. Finally, 11 possible regulatory pathways and potential targeted medicines of the signature gene were uncovered.

Spliceosome disorders are closely related to the development and progression of cancer. Small molecule inhibitors and other novel therapies that target spliceosomes or their cofactors could become new options for cancer treatment [16, 17]. The molecules B/D1/D2/D3/E/F/G are the core Sm proteins that make up splice snRNPs and have a significant role in the development of cancer, according to previous research [18]. SNRPB is significantly linked to cisplatin resistance in NSCLC, and overexpressed SNRPBs enhance the inhibitory effect of cisplatin on H460 cell-mediated xenografted tumors [19, 20]. SNRPB can mediate RNA splicing and drive the proliferation and dryness of tumor cells in hepatocellular carcinoma. It is interesting that we discovered that SNRPB plays a crucial role in LUAD bioinformatics prediction as a splicing factor for core carcinogenesis. We discovered that the central components of the protein interaction network diagram are SNRPB, SNRPE, and SNRPN. In the network diagram of drug mining, we discovered that SNRPB interacts with anticancer medications such sunitinib, arsenic trioxide, and copper in addition to the reported cisplatin. This finding may be helpful in determining the best pharmacological therapies for LUAD patients.

We discovered that SNRPD2 was also included as a critical signature gene while creating LUAD prognostic signatures. SNRPD1, SNRPD3, and SNRPD2 have been identified as spliceosome-related proteins that have previously been crucial in the genesis of cancer. There are few studies on the clinical and prognostic effects of the SNRPD1 gene; however, some research on SNRPD1 in malignancies mostly focused on the discovery of molecular biological mechanisms [21–23]. Recent studies have found that SNRPD2 and SNRPD3, as RNA splicing factors, can inhibit the proliferation of breast cancer cells and laryngeal squamous carcinoma cells and participate in the study of cell cycle-related mechanisms during early fracture [24, 25].

Furthermore, our results suggested that SNRPE may play an important role in immune checkpoint inhibitor therapy. By analyzing the correlation results between 11 signature genes and immune checkpoint genes, we found that only SNRPE was significantly negatively correlated with 8 molecules such as CD274 and CTLA4. The study found that SNRPE is mainly involved in the disease progression of tumors such as hepatocellular carcinoma, adrenocortical carcinoma, lung cancer, and multiple myeloma [26–29]. However, whether splicing factors such as SNRPE affect immunotherapy and molecular mechanisms of LUAD has not been reported. Therefore, our immunologic exploration of spliceosome genes is of great significance for improving the efficacy of immunotherapy in patients.

In our finding, five members of the LSM family, LSM2, LSM4, LSM5, LSM7, and LSM8, were incorporated into our LUAD prognosis model and drug mining network. Previous studies have shown that LSM2 plays a role in the disease progression of lung cancer [30]. Hua et al. also found that LSM2 may be associated with the prognosis of ovarian cancer and may be involved in influencing the response to treatment with platinum-based drugs [31]. Abnormal expression of LSM4 is closely related to the occurrence and development of a variety of tumors. LSM4 has been found to affect the production and movement of tumor cells in esophageal cancer [32]. LSM4 is involved in the disease progression of pancreatic cancer by mediating the formation of U4/U6 snRNP [33, 34]. Recent research by Chen et al. suggested that LSM4 may affect the growth and transfer of HCC cells by regulating key pathways such as cell cycles, adhesive plaques, and metabolic-related pathways [35]. At present, the mechanism of action of LSM4 and LSM5 in lung cancer has not been reported. A previous study has shown that LSM7 is significantly associated with T cell responses in breast cancer [36]. However, our study found that LSM7 in LUAD was only associated with 3 immune checkpoint molecules such as PDCDILG2, LAG3, and TIGIT. This may be related to the heterogeneity of the tumor. The study by Li et al. found that the RNA-binding protein LSM7 is also associated with the prognosis of LUSC and has application value for personalized treatment of patients [37]. In addition, our study innovatively predicts the TF-hub network of spliceosome-related genes, providing reference value for further mechanistic studies. Star transcription factors such as TP53, ZEB1, and SIRT6 have been reported to play an important role in areas such as tumor resistance, metastasis, and tumor metabolism [38–40].

In conclusion, this study is the first to examine the importance of genes connected to spliceosomes in the diagnosis and care of LUAD. We have provided novel therapeutic medication targets and prospective prognostic genes for the treatment of LUAD patients through our study. Furthermore, in our future research, we should make use of more animal models and clinical trials to investigate the role of these possible target spliceosome genes in the prognosis of LUAD.

## Data Availability

All data was obtained from the public database described in Materials and Methods.
